# Advancing Health Equity of Older Adults Through a Community-Engaged Recreational Needs Assessment in Philadelphia

**DOI:** 10.1093/geroni/igaf122.929

**Published:** 2025-12-31

**Authors:** Anna Grasso, Laura Baehr

**Affiliations:** Drexel University, Philadelphia, Pennsylvania, United States; Drexel University, Philadelphia, Pennsylvania, United States

## Abstract

Most older adults are living with at least one chronic health condition that can be positively modified through regular exercise, but less than 15% of this population achieve national physical activity guidelines for health. Several social, organizational, and environmental barriers contribute to the physical activity participation gap. Physical inactivity is highest among older adults with chronic disease and disability, reporting non-white race or Hispanic ethnicity, and lower socioeconomic and educational levels. Inaccessible recreational environments also limit community-based exercise and physical activity by older adults. While it is a federal mandate for all public buildings to comply with Americans with Disabilities Act (ADA) accessibility standards, there are additional recreational considerations for older adults that are rarely addressed or corrected. The purpose of this project is to evaluate the recreational accessibility of the Drexel Recreation Center, a public health club in the heart of an urban university campus that is proximate to some of the most socioeconomically disadvantaged older adult residents in the United States as defined by the Area Deprivation Index. Using the Person-Environment-Occupation-Performance (PEOP) model, the team completed a recreational needs assessment inclusive of the Accessibility Instruments Measuring Fitness and Recreation Environments (AIMFREE) tool, physical therapy and occupational therapy clinical knowledge, and community advisory board member input. A voluntary electronic survey was sent to older adults who are current or prospective members of the recreation center to determine use, barriers, and facilitators to participation. Data collection is ongoing, with a planned townhall to distribute results to all invested community members.

